# “Salicylic acid enhances thermotolerance and antioxidant defense in *Trigonella foenum graecum* L. under heat stress”

**DOI:** 10.1016/j.heliyon.2024.e27227

**Published:** 2024-03-11

**Authors:** Sana Choudhary, Towseef Mohsin Bhat, Khairiah Mubarak Alwutayd, Diaa Abd El-Moneim, Neha Naaz

**Affiliations:** a Cell biology, molecular Biology and Genetics section, Department of Botany, Aligarh Muslim University, Aligarh, 202002, India; b Government of Education, Jammu and Kashmir, India; c Department of Biology, College of Science, Princess Nourah bint Abdulrahman University, Riyadh, 11671, Saudi Arabia; d Department of Plant Production (Genetic Branch), Faculty of Environmental Agricultural Sciences, Arish University, El-Arish, Egypt

**Keywords:** Enzymes, High temperature stress, Proteins, Salicylic acid, *Trigonella foenum-graecum*

## Abstract

Plants possess various defense mechanisms to cope with genotoxic and environmental challenges, with high temperatures posing a significant threat due to global warming. In this investigation, ten-day-old *Trigonella foenum-graecum* (fenugreek) seedlings were cultivated in a controlled environment chamber with conditions set at 70–80% relative humidity, a day/night cycle of 25/18 °C, and a photosynthetically active radiation (PAR) of 1000 μmol m^−2^ s^−1^. Other groups of seedlings were subjected to temperatures of 30, 35, or 40 °C. Our research aimed to investigate the relationship between temperature intensity, duration, growth responses, physiological and metabolic activities, and the stress alleviation by salicylic acid. The results demonstrated that high temperatures significantly reduced plant growth, membrane stability, while increasing proline and protein content, as well as electrolyte leakage in the leaves. The most pronounced results were observed when exposed to 40 °C for 24 h. Salicylic acid completely mitigated the negative impacts of high-temperature stress when it was applied at 40 °C for 24 h. We utilized two-dimensional electrophoresis and matrix-assisted laser desorption/ionization time-of-flight mass spectrometry to examine proteins across three groups: control plants, stressed plants, and plants subjected to salicylic acid treatment. Our results revealed that, among the proteins influenced by high-temperature stress, 12 displayed the most significant differences in regulation. These stress-responsive proteins played roles in signal transduction, stress defense, detoxification, amino acid metabolism, protein metabolism (including translation, processing, and degradation), photosynthesis, carbohydrate metabolism, and energy pathways. These proteins may hold practical implications for diverse biological activities. In conclusion, salicylic acid treatment enhanced thermotolerance in fenugreek plants, although further investigation is required at the genome level to elucidate the mechanism of salicylic acid action under heat stress.

## 1 Introduction

The contentious issue of global warming, primarily driven by an increasing population and human activities, stems from the contentious matter of fossil fuel consumption. This phenomenon leads to significant alterations in climate patterns, posing severe threats to food security [1]. The key driver of global warming is the heightened concentrations of greenhouse gases. High-temperature stress is typically characterized by an increase in temperature intensity that is significant enough to result in permanent harm to the growth and development of plants. Heat stress (HS) has emerged as a substantial factor constraining crop production and global food security. As a result, it is expected that the average annual temperatures worldwide will increase within the range of 0.3–4.8 °C by the year 2100 [2]. Elevated temperatures present a worldwide agronomic challenge. Stress induced by either brief or prolonged high temperatures induces morphological, anatomical, and physio-biochemical alterations in plants, negatively impacting their growth as well as development and resulting in a significant decline in economic output [3]. Additionally, climate change is anticipated to result in more frequent and intense heatwaves [4], consequently diminishing the chances of plant survival, impeding growth, and reducing crop yields [5,6].

Plants being immobile are more susceptible to get affected by heat stress [7] with a significant disruption in various activities. The influence of heat stress on plants is substantial, affecting various activities such as seed germination, growth, development, photosynthesis, and reproduction, ultimately leading to significant repercussions on overall plant growth as well as yield [8,9]. Responding to heat stress, plants undergo morphological changes, including an increase in the root system, a reduction in stomatal number and conductance, and curling, folding, and thinning of leaves to minimize water loss through evapotranspiration [10,11]. Heat stress's negative effects can be mitigated by developing thermo-tolerant plants employing a variety of physiological and genetic techniques. Accordingly, a detailed insight of mechanisms of heat tolerance, physiological reactions of plants to high temperatures, and potential approaches for enhancing crop productivity under stress conditions is crucial [[12], [13], [14], [15], [16]]. Under high temperatures, several physiological as well as biochemical mechanisms are activated in plants which are imperative to get through stress [17]. High temperature during early growth slows down or inhibits germination dependent on species of the plant and the stress potency [18]. Primary injuries owing to high temperatures induce protein denaturation as well as aggregation, boosted fluidity of membrane lipids, and secondary heat damages involve reduction in protein synthesis, protein degradation as well as loss of membrane integrity [19].

The molecular response of plants to abiotic stresses is frequently investigated as a intricate process primarily centred on the modulation of the transcriptional activity of genes associated with stress [20], and antioxidative biomarkers induce an adaptive reaction to high-temperature stress. To adapt to heat stress, the antioxidant defence mechanism is crucial, and the degree to which it is active is related to the advancement of thermotolerance [21,22]. The primary challenge posed by heat stress is the opening of stomata, which aims to lower leaf temperature while increasing transpiration, respiration, and photosynthesis. Consequently, the concurrent application of heat stress affects gas exchange signals [23]. Additional detoxification mechanisms are triggered, notably the heightened activity of antioxidant enzymes and the synthesis of heat shock proteins (HSPs). Moreover, the mechanisms that plants have developed to mitigate the harm triggered by high-temperature stress are linked to distinct stress signaling molecules like salicylic acid, jasmonic acid, and ethylene [24]. These compounds played a role in the cellular response to elevated temperatures induced by abiotic stress [25].

A naturally occurring phenolic substance and endogenous signal molecule, salicylic acid (SA), is essential for controlling plant development, growth, interactions with other plant hormones, and responses to environmental stress [26,27]. Because SA plays a role in plant defence systems against biotic and/or abiotic stress, it may also help plants cope with high temperatures [[28], [29], [30], [31], [32]]. Earlier reports on the exogenous application of SA show that it affects a broad array of physiological processes, involving improved tolerance of salinity in *Hordeum vulgare* [33], drought stress in *Ocimum basilicum* [34], salinity and drought stress in *Mentha pulegium* [35], water stress in *Cucurbita pepo* [36], heat stress in *Medicago sativa* [37], salinity stress in *Trigonella* [38], and also reduced in the severe outcomes of drought stress on cucumber [39].

Even though there have been several reports on the biochemical and molecular events arising in high-temperature stress, fewer data have been found showing the alleviation of thermotolerance by exogenous application of salicylic acid against high temperature (Heat stress). Various plant species and varieties demonstrate a broad spectrum of plasticity in heat tolerance, spanning from high sensitivity to the hyper-accumulation of several crucial biomolecules. Leguminous plants, including *T. foenum-graecum*, are less tolerant to high temperatures (heat stress) than cereals and grasses [40]. Fenugreek (*T. foenum-graecum* L.) is a multi-purpose crop widely cultivated throughout most regions worldwide for its medicinal properties [38]. Fenugreek harbors numerous bioactive phytochemicals, including trigonelline (alkaloid), diosgenin (steroidal sapogenin), and mucilage, renowned for their tasks in plant protection [41,42]. In India and other Asian countries, fenugreek seeds are extensively utilized in culinary applications owed to their robust flavor as well as diverse biological tasks, including gastroprotective, anticancer, hypocholesterolemic, and hypoglycemic properties [43,44].

We have aimed to examine the outcome of exposure of fenugreek plants to high temperatures throughout the initial phases of their formation and its impact on the physiological and biochemical assets of its leaves. This analysis primarily concentrates on the processes by which SA alleviates tolerance in fenugreek seedlings to high-temperature stress and alleviates the responsible proteins involved in this pathway.

## 2 Material and methods

### 2.1 Hormone preparation

To prepare stock solutions of salicylic acid (SA), the necessary amount of SA was dissolved in 5 ml of ethanol in 100 ml volumetric flasks. Subsequently, 5 mL of the surfactant "Tween-20″ was introduced, and the final volume was adjusted to the mark via double-distilled water (DDW). Dilution of the stock solution with DDW was performed to achieve a concentration of 0.20 mM for SA.

### 2.2 Germplasm procurement, treatment, and maintenance of plant material

Certifiable seeds of *T. foenum-graecum* L. cv. Azad (Thermo-tolerant, high-yielding cultivar) was acquired from the National Bureau of Plant Genetic Resources (NBPGR), New Delhi, India. A 0.01% mercuric chloride solution was used to surface sterilize the healthy seeds, and the adhering mercuric chloride solution was removed by repeatedly washing the seeds in DDW. This experimentation was carried out in October–December 2012 with 40 earthen pots (12-inch pots filled with autoclaved soil equally) with five replicates for each treatment. Three seedlings were kept in every pot in a simple randomized block design. Ten-day-old fenugreek seedlings were placed in a greenhouse with a 70–80% relative humidity, a day/night cycle of 25/18 °C, and maximum photosynthetically active radiation at approximately 1000 μmol photons m^2^ s^−1^. Fenugreek with uniform growth (10 leaves) underwent a two-day acclimatization period in a controlled environment room (70–80% relative humidity, 25/18 °C day/night cycle, and 800 μmol/m^2^ s^−1^) before being split into four groups. One group served as a control and was maintained at 25 °C. The other groups were subjected to heat stress at 30, 35, or 40 °C for 12 h (T_1_, T_2_, and T_3_, respectively) and 24 h (T_4_, T_5_, and T_6_, respectively) in controlled environment rooms, with conditions identical to the 25 °C room except for temperature. Various combination treatments involved the exogenous application of salicylic acid (SA) under different heat stress conditions, labeled as T_1_+SA, T_2_+SA, T_3_+SA, T_4_+SA, T_5_+SA, and T_6_+SA. Following the heat stress treatments, the stressed plants were allowed to recover at 25 °C, maintaining the same environment as before the heat treatments, in the net house of the Botany department at Aligarh Muslim University, Aligarh, India (27.88 N 78.08E) under natural environmental conditions. After ten days, the plants were sprayed with SA (0.20 mM) (excluding controls). Each seedling received three sprinklings, with the sprayer nozzle adjusted to dispense 1 ml of double-distilled water (DDW) or SA solution in one sprinkle. The plants were then sampled at 30 days after sowing (DAS) to evaluate various parameters.

### 2.3 Plant growth parameters

#### 2.3.1 Determination of growth

Growth was assessed using parameters including germination (%), shoot length, root length, fresh weight, and dry weight. A ruler assessed the elongation of roots as well as shoots. The calculation was based on the length of the roots and shoots from not less than three separate seedlings from every treatment level. Using a Metler Toledo precision balance that is precise to three decimal places, tissue sections were blotted on Whatman paper, dried at 70 °C, and balanced. Dry weight was analyzed after proper hydration using the method described in Ref. [45].

#### 2.3.2 Antioxidant enzyme activity

To analyze antioxidant enzyme activities, a leaf fresh mass of 0.5 g was homogenized with 5 mL of 50 mM phosphate buffer (pH 7.0) and 1% PVP (polyvinyl pyrrolidone). The resulting homogenate underwent centrifugation at 10,000×*g* for 10 min at 4 °C, and the supernatant was used for antioxidant enzyme analysis, such as Superoxide Dismutase (SOD), Catalase (CAT), Peroxidase (POD), and Glutathione Reductase (GR). For POD activity, 50 μL of enzyme extract was added to a reaction mixture containing 1.0 mL of 50 mM sodium phosphate (pH 5.5), 1.0 mL of 0.3% H_2_O_2_, and 0.95 mL of 0.2% guaiacol. The absorbance changes at 470 nm were recorded as a measure of POD enzyme activity, based on its ability to convert guaiacol to tetraguaiacol [46]. CAT activity was determined by adding 200 μL of enzyme extract to a reaction mixture consisting of 1.5 mL of 50 mM sodium phosphate (pH 7.8), 300 μL of 0.1 M H_2_O_2_, and 1.0 mL of distilled water. The reduction of H_2_O_2_ was monitored, and the change in absorbance at 240 nm per minute represented CAT activity [47]. SOD activity was determined according to Beauchamp and Fridovich [48]. In this method, 0.1 mL of enzyme extract was added to a reaction mixture containing 1.5 mL of 50 mM sodium phosphate (pH 7.8), 0.3 mL of 130 μM methionine, 0.3 mL of 750 μM nitro-blue tetrazolium (NBT), 0.3 mL of 100 μM EDTA- Na_2_, 0.300 mL of 20 μM riboflavin, and 100 μL of distilled water. After illumination under light at 4000 flux for 20 min, the sample's absorbance was measured at 560 nm. SOD activity was expressed as the amount of enzyme needed for 50% inhibition of NBT reduction. GR activity was estimated by monitoring the oxidation of NADPH at 340 nm, following the method of Schaedle and Bassham [49]. The procedures for all these antioxidant enzyme activities were revised from Ref. [50].

### 2.4 Biochemical parameters

#### 2.4.1 Chlorophyll content

The chlorophyll content in the entire leaves of the plants (one plant from each replicate) was measured using a SPAD chlorophyll meter (Minolta 502) [51].

#### 2.4.2 Leaf electrolytic leakage (EL)

To evaluate membrane stability and assess the relative ion content in the apoplastic space, electrolyte leakage was determined following the method outlined by Sullivan and Ross [52]. The calculation of electrolyte leakage was performed using the provided Eq. (1):

(1) $$Electrolyte\;Leakage\left(\%\right)=\frac{ECb-ECa}{ECc}\times 100$$

#### 2.4.3 Lipid peroxidation and H_2_O_2_ content

The degree of membrane lipid peroxidation was determined by quantifying malondialdehyde (MDA), a decomposed product originating from peroxidized polyunsaturated fatty acids in the membrane lipid composition. This assessment utilized thiobarbituric acid (TBA) as the reactive substance, following the procedure outlined by Heath and Packer [53]. For the analysis, a leaf sample weighing 0.25 g was homogenized in 5 mL of 5% trichloroacetic acid (TCA), and the resulting homogenate underwent centrifugation for 5 min at 10,000×*g*. After centrifugation, the supernatant was combined with thiobarbituric acid (2 mL), and the reaction mixture was heated for 30 min at 95 °C. Following incubation, the mixture was cooled to room temperature, and absorbance was measured at 532 and 600 nm. The concentration of MDA was calculated using the extinction coefficient of 155 mM^−1^ cm^−1^ and expressed as nmol of MDA g^−1^ fresh weight.

For hydrogen peroxide (H_2_O_2_) content determination, a leaf sample weighing 0.25 g was extracted with ice-cold TCA (5%) and centrifuged for 5 min at 10,000×*g*. Subsequently, 500 μL of the supernatant was mixed with 500 μL of 50 mM potassium phosphate buffer (pH 7.5) and potassium iodide (1 mL). The reaction mixture was incubated for 20 min at room temperature, and absorbance was recorded at 390 nm to quantify the H_2_O_2_ content [54].

#### 2.4.4 Soluble protein estimation

Protein quantification was conducted following the method outlined by Bradford [55]. Leaf samples were homogenized in 0.1 M Na-phosphate buffer at pH 7. Prior to extraction, 10 mg of insoluble polyvinyl pyrrolidine was added to the tissue samples. The resulting homogenates underwent centrifugation at 20,000 rpm at 4 °C for 15 min. The obtained supernatants were then subjected to a second centrifugation for 60 min at 20,000 rpm at 4 °C. Absorbance was measured at 595 nm, and the concentration was calculated using bovine serum albumin as a standard.

### 2.5 Proteomic analysis

#### 2.5.1 Extraction of whole protein from treated fenugreek seeds after 45 DAS of sowing

The method for extracting whole proteins, as outlined in Refs. [56,57], was utilized with minor modifications, using the TCA-Acetone precipitation technique.

#### 2.5.2 First*-*dimension isoelectric focusing (IEF)

The initial step of isoelectric focusing (IEF) was conducted using 17 cm pH 4–7 linear immobilized pH gradient (IPG) strips in the Ettan IP Gphor III IEF System from GE Healthcare. Active rehydration was performed, and the rehydration solution consisted of 450 μL rehydration buffer (6 M urea, 2 mol L^−1^ thiourea, 2% CHAPS, 0.5% IPG buffer, 0.002% bromophenol blue) containing 1000 μg of protein and dithiothreitol (DTT). Isoelectric focusing was carried out at voltages of 500 V for 1 h, 1000 V for 1 h, and 8000 V for a total of 14.5 kVh.

#### 2.5.3 Second-dimension SDS-PAGE

Following the completion of isoelectric focusing (IEF), the gel strips were incubated with equilibration buffer I (50 mmol L^−1^ Tris–HCl, (pH 8.7), 6 M urea, 30% glycerol, 2% (m/v) SDS, 0.002% bromophenol blue, 1% DTT). Subsequently, the strips were re-equilibrated for 15 min in equilibration buffer II (3.5 M Tris–HCl, pH 8.0, 6 M urea, 30% glycerol, 2% SDS, 0.003% bromophenol blue, 2% iodoacetamide) each on a shaker. After decanting the equilibration buffer, the strips were washed with water and placed on a 12% polyacrylamide gel (8 × 13 cm) using a Tris-glycine buffer system. The strips were overlaid with an agarose sealing solution, and the SE 600 Ruby Standard Dual Cool Vertical electrophoresis Unit (Amersham Biosciences) was employed. Visualization of the 2D-PAGE gels were accomplished by staining with Coomassie Brilliant Blue G-250 (colloidal). The gels were fixed overnight in a solution of 50% ethanol and 10% acetic acid, followed by two 20-min washes with deionized water. Subsequently, the gels were immersed for 1 h in a solution containing 34% methanol, 17% ammonium sulfate, and 3% phosphoric acid, and then stained in the same solution with Coomassie Blue G-250 (0.066%) for 2 days. Afterward, the gels were washed with distilled water and stored in a 20% ammonium sulfate solution. Gel images were captured using a Gel Doc 2000 (Bio-Rad) image scanner, and spot detection, spot matching, and quantitative intensity analysis were conducted using Melanie 7.0 software (Gene Bio).

#### 2.5.4 In-gel digestion of diﬀerentially expressed proteins

The protein spots of interest were extracted from the gels by tryptic digestion, and sample preparation was achieved according to Refs. [58,59] and with minimal adjustments.

#### 2.5.5 MALDI-TOF-TOF/MS and data analysis

Following the experimental procedures, samples underwent analysis using a 4800 Proteomics Analyzer (MALDI-TOF/TOF-TM) (ABI Sciex 5800). After drying, the peptides were dissolved in 0.6 μL of 0.4 g L^−1^ CHCA solution (0.1% TFA + 50% CAN solvent). The solution was air-dried at room temperature and subsequently identified on the MALDI target plate. Mass spectrometric analysis was then performed on the samples using a Nd:YAG laser with a wavelength of 355 nm and an accelerating voltage of 20 kV. Data collection was conducted in positive ion and automated acquisition mode. The PMF mass scan ranged from 700D to 3500D, and series mass spectrometric analysis was performed on the five peaks with the maximum intensity. The myoglobin enzymolysis peptide segment on the spectrogram was used for standard external adjustment. The monoisotopic peptide masses obtained from MALDI-TOF-TOF were analyzed using the 4000 Series Explorer software version 3.5 (ABI). For protein identification, the mass signals were searched against NCBI and Swiss Prot databases using the BLAST (Basic Local Alignment Search Tool) software.

### 2.6 Statistical analysis

Each treatment was replicated five times for a comprehensive analysis. Statistical analysis of the data was carried out using SPSS 17.0 for Windows (SPSS, Chicago, IL, USA). To identify statistically significant differences between the control and treatment groups, a one-way analysis of variance (ANOVA) was performed. Subsequently, Dunnett's multiple comparison tests were employed to further analyze significant differences identified in the ANOVA.

## 3 Results

### 3.1 Growth parameters (germination, shoot and root length)

**Fig. 1:**
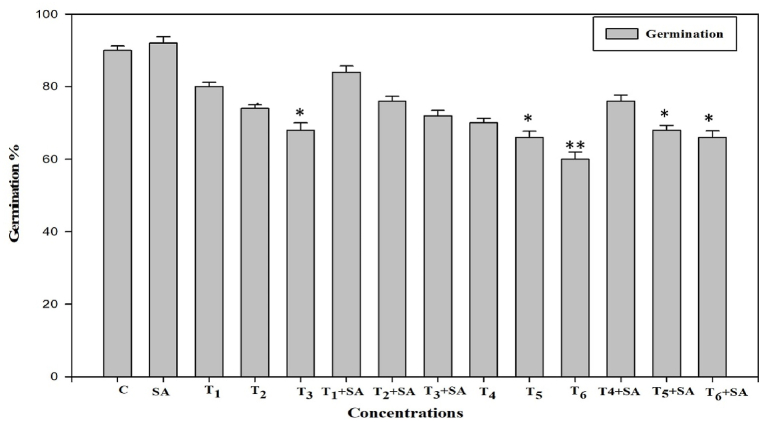
Response of Salicylic acid (0.20 mM) on the high temperature (30, 35 and 40 °C) induced changes in germination in *Trigonella* at 30 days stage if growth [C= Control, SA= Salicylic acid, T1 = 30 °C, T2 = 35 °C, T3 = 40 °C for 12h and T4 = 30 °C, T5 = 35 °C, T6 = 40 °C for 24h of heat stress] Vertical bar shows standard errors and values with (*) and (**) differ significantly at p < 0.05 and p < 0.01, respectively using DMRT.

**Fig. 2:**
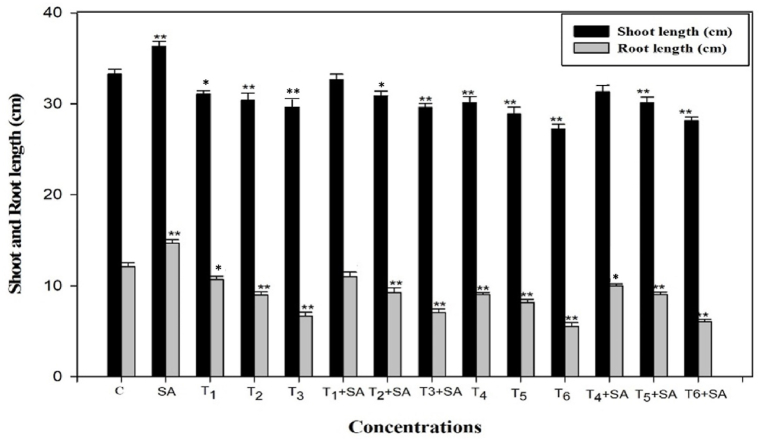
Response of Salicylic acid (0.20 mM) on the high temperature (30, 35 and 40 °C) induced changes on shoot and root length in *Trigonella* at 30 days stage if growth [C= Control, SA= Salicylic acid, T_1_ = 30 °C, T_2_ = 35 °C, T_3_ = 40 °C for 12h and T_4_ = 30 °C, T_5_ = 35 °C, T_6_ = 40 °C for 24h of heat stress] Vertical bar shows standard errors and values with (*) and (**) differ significantly at p < 0.05 and p < 0.01, respectively using DMRT.

Heat stress negatively affected plant growth, shoot and root length of fenugreek. Plants subjected to 40 °C heat stress were severely affected. Compared to control, high temperature stress (40 °C) showed significant reduction in germination by 33.33%, root length by 54.20% and shoot length by 18.09% compared with the control temperature (25 °C). Exogenous supplementation of SA effectively improved all growth parameters by 2.22%, 21.15%, and 9.10% for germination, root length, and shoot length, respectively under high temperature stress. However, the treatment with SA alone (0.20 mM) under normal temperature had no significant effect on germination compared to control while had significant effect (*p* < 0.01) on shoot length and root length. The combination treatments T_5_+SA and T_6_+SA showed significant increase (*p* < 0.05) in the germination pattern. Similarly, shoot and root length was significantly increased in the combination treatments. The germination, shoot and root length were longer in the high temperature-stressed plants given hormone treatments than in the high temperature-stressed plants without hormone treatments. Though, the follow-up treatment with SA (0.20 mM) to some extent overcomes the detrimental effect created with temperature (Fig. 1, Fig. 2).

### 3.2 Plant biomass (shoot and root dry weight)

**Fig. 3:**
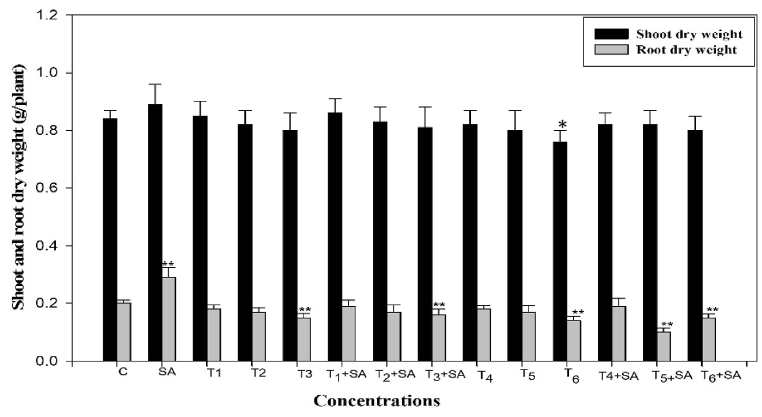
Response of Salicylic acid (0.20 mM) on the high temperature (30, 35 and 40 °C) induced changes on shoot and root dry weight in *Trigonella* at 30 days stage if growth [C= Control, SA= Salicylic acid, T_1_ = 30 °C, T_2_ = 35 °C, T_3_ = 40 °C for 12h and T_4_ = 30 °C, T_5_ = 35 °C, T_6_ = 40 °C for 24h of heat stress] Vertical bar shows standard errors and values with (*) and (**) differ significantly at p < 0.05 and p < 0.01, respectively using DMRT.

A remarkable decrease in plant biomass (Shoot dry weight and Root dry weight) with increasing temperature stress was observed. The intensity of inhibition in root length was 1.17%, 4.65%, and 6.97% (in 30, 35, and 40 °C for 12h) and 4.65%, 6.97% and 11.60% (in 30, 35 and 40 °C for 24h of heat treatment) less than their respective control. Shoot length was also significantly decreased (*p* < 0.01) when plants were exposed to elevated temperatures. The highest temperature (40 °C for 24h) proved more inhibitory than the lower temperature stress (40 °C for 12h). The application of SA (0.20 mM) alone significantly (*p* < 0.01) increased root length by 21.12% and non-significantly increase the shoot length by 9.10%, over the control (Fig. 3). The application of SA neutralized the injury produced by heat stress in all treatments. However, there is no significant increase in shoot length when exposed to the combination treatments of heat stress along with SA, while significantly (*p* < 0.01) increased the root length.

### 3.3 Antioxidant enzyme activity

**Fig. 4:**
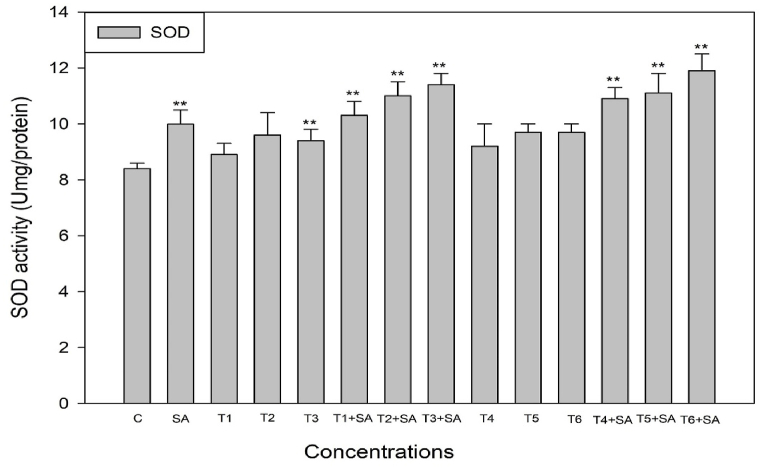
Response of Salicylic acid (0.20 mM) on the high temperature (30, 35 and 40 °C) induced changes on Superoxide dismutase enzyme activity in *Trigonella* at 30 days stage if growth [C= Control, SA= Salicylic acid, T_1_ = 30 °C, T_2_ = 35 °C, T_3_ = 40 °C for 12h and T_4_ = 30 °C, T_5_ = 35 °C, T_6_ = 40 °C for 24h of heat stress] Vertical bar shows standard errors and values with (*) and (**) differ significantly at p < 0.05 and p < 0.01, respectively using DMRT.

**Fig. 5:**
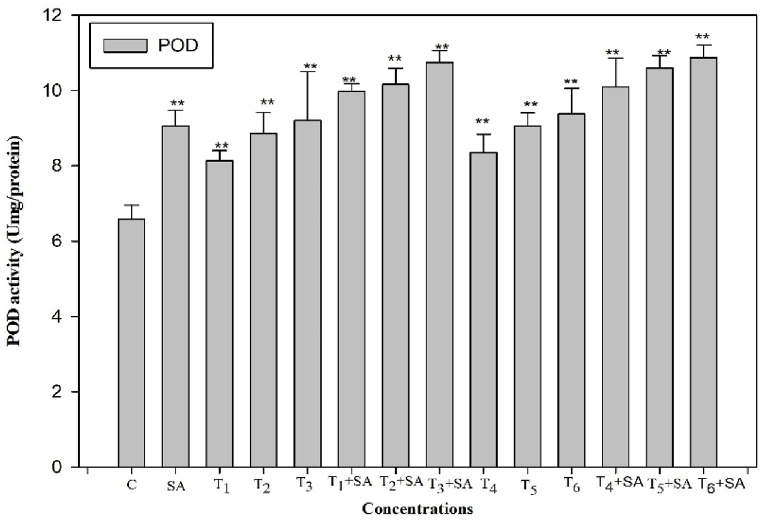
Response of Salicylic acid (0.20 mM) on the high temperature (30, 35 and 40 °C) induced changes on peroxidase enzyme activity in *Trigonella* at 30 days stage if growth [C= Control, SA= Salicylic acid, T_1_ = 30 °C, T_2_ = 35 °C, T_3_ = 40 °C for 12h and T_4_ = 30 °C, T_5_ = 35 °C, T_6_ = 40 °C for 24h of heat stress] Vertical bar shows standard errors and values with (*) and (**) differ significantly at p < 0.05 and p < 0.01, respectively using DMRT.

**Fig. 6:**
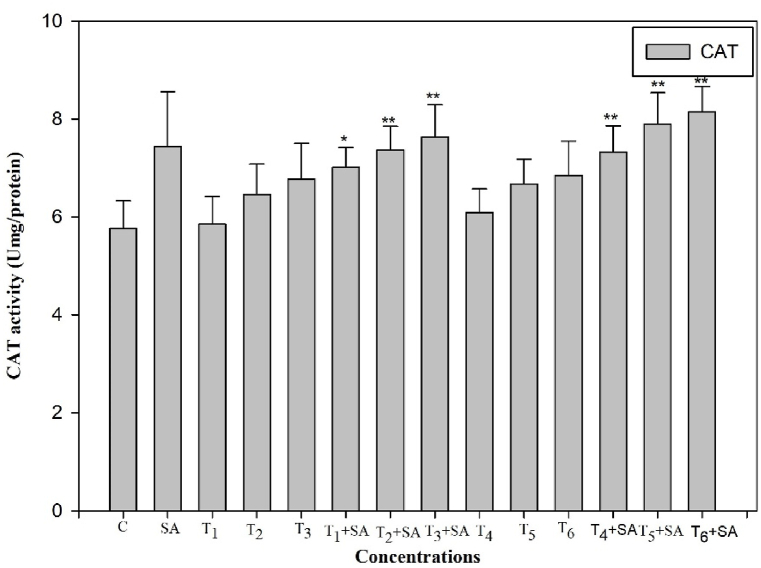
Response of Salicylic acid (0.20 mM) on the high temperature (30, 35 and 40 °C) induced changes on catalase enzyme activity in *Trigonella* at 30 days stage if growth [C= Control, SA= Salicylic acid, T_1_ = 30 °C, T_2_ = 35 °C, T_3_ = 40 °C for 12h and T_4_ = 30 °C, T_5_ = 35 °C, T_6_ = 40 °C for 24h of heat stress] Vertical bar shows standard errors and values with (*) and (**) differ significantly at p < 0.05 and p < 0.01, respectively using DMRT.

**Fig. 7:**
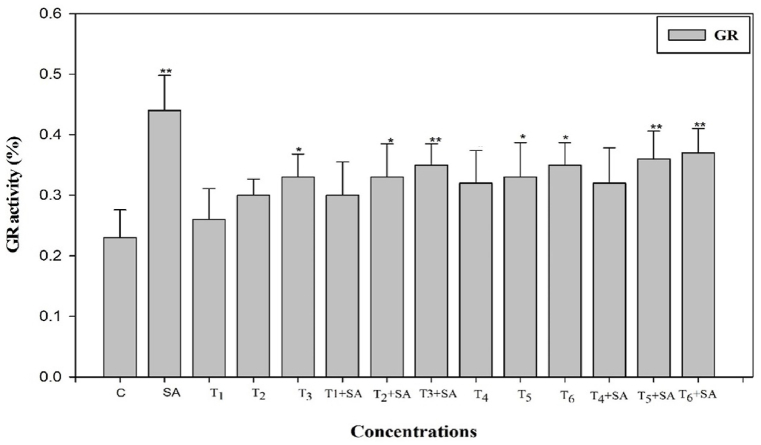
Response of Salicylic acid (0.20 mM) on the high temperature (30, 35 and 40 °C) induced changes on glutathione reductase activity in *Trigonella* at 30 days stage if growth [C= Control, SA= Salicylic acid, T_1_ = 30 °C, T_2_ = 35 °C, T_3_ = 40 °C for 12h and T_4_ = 30 °C, T_5_ = 35 °C, T_6_ = 40 °C for 24h of heat stress] Vertical bar shows standard errors and values with (*) and (**) differ significantly at p < 0.05 and p < 0.01, respectively using DMRT.

To ascertain whether salicylic acid (SA) plays a role in alleviating oxidative stress induced by heat stress through reactive oxygen species (ROS) scavenging, we examined the activity of antioxidant enzymes following SA treatment, with or without subjecting the plants to high-temperature stress. The exogenous application of SA demonstrated a positive and increased effect on the activities of these enzymes when plants were exposed to heat stress. The activities of antioxidant enzymes (SOD, POD, CAT, and GR) were significantly elevated (*p* < 0.05, 0.01) when plants were subjected to high-temperature stress alone and in combination treatments (Fig. 4, Fig. 5, Fig. 6, Fig. 7). Upon exposure to 40 °C for 24 h, plants exhibited increased percentages of SOD, POD, and CAT by 16.50%, 42.85%, and 18.92%, respectively, compared to the control. The activity of SOD, POD, and CAT also showed enhancement in plants treated with SA without heat stress compared to the control. Additionally, when plants were exposed to the same degree of heat stress along with SA treatment, the maximum activity of SOD, POD, and CAT increased by 44.86%, 65.19%, and 41.49%, respectively, compared to normal growth conditions. The control plant had the least value of these enzymes (Fig. 4, Fig. 5, Fig. 6).

The plant treated with SA alone possessed maximum values of GR was about 91.30% higher over the control. The activity of GR was also found to significantly increase (*p* < 0.05, 0.01) as compared to control in the plants subjected to all temperature stress. Still, the maximum increase, i.e., 60%, was recorded at a higher temperature 40 °C, for 24h along with SA over the control. It is evident from Fig. 7.

### 3.4 Protein content

**Fig. 8:**
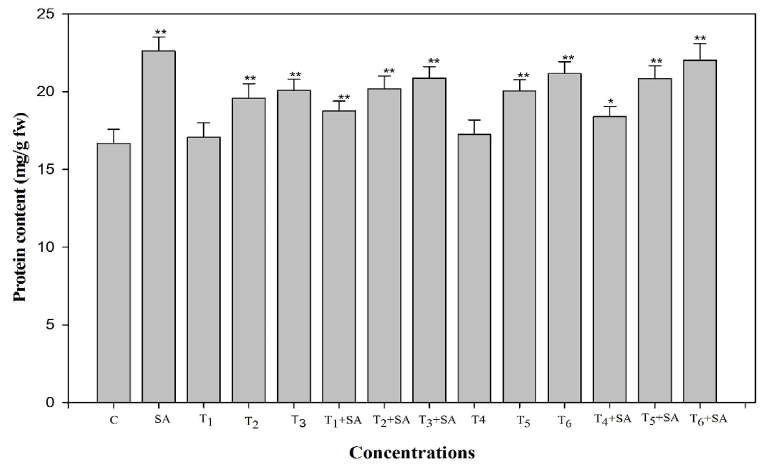
Response of Salicylic acid (0.20 mM) on the high temperature (30, 35 and 40 °C) induced changes on protein content in *Trigonella* at 30 days stage if growth [C= Control, SA= Salicylic acid, T_1_ = 30 °C, T_2_ = 35 °C, T_3_ = 40 °C for 12h and T_4_ = 30 °C, T_5_ = 35 °C, T_6_ = 40 °C for 24h of heat stress] Vertical bar shows standard errors and values with (*) and (**) differ significantly at p < 0.05 and p < 0.01, respectively using DMRT.

A profound and significant increase in the protein content was recorded due to the high temperature stress. Heat stress induced a notable increase (*p* < 0.05, 0.01) in protein levels by 2.39%, 17.03%, and 20.45% at 30, 35, and 40 °C for 12 h and 3.47%, 20.27%, and 26.93% at 30, 35, and 40 °C for 24 h, respectively, compared to the unstressed control. When salicylic acid (SA) was applied exogenously, a separate treatment exhibited the maximum increase, reaching 35.63%. Furthermore, heat-stressed plants treated with SA displayed significant enhancements in protein content compared to untreated heat-stressed plants. Notably, plants treated with the combination of SA and high temperature (40 °C for 24 h) maintained a maximum increase of 32.03% compared to plants treated with either T + SA or SA alone (Fig. 8).

### 3.5 Chlorophyll content

**Fig. 9:**
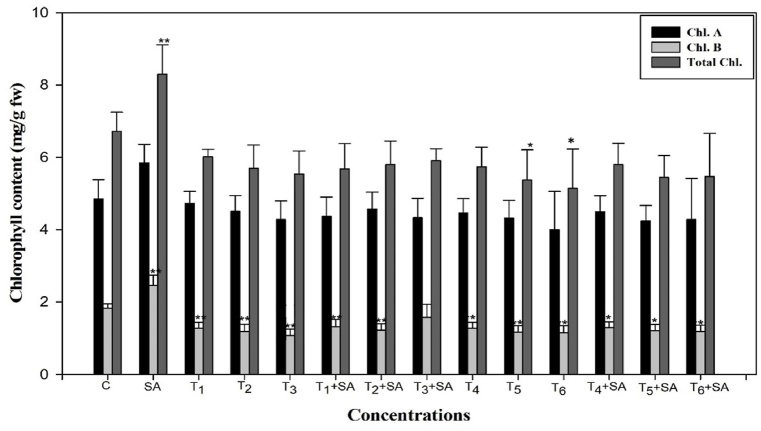
Response of Salicylic acid (0.20 mM) on the high temperature (30, 35 and 40 °C) induced changes on chlorophyll content in *Trigonella* at 30 days stage if growth [C= Control, SA= Salicylic acid, T_1_ = 30 °C, T_2_ = 35 °C, T_3_ = 40 °C for 12h and T_4_ = 30 °C, T_5_ = 35 °C, T_6_ = 40 °C for 24h of heat stress] Vertical bar shows standard errors and values with (*) and (**) differ significantly at p < 0.05 and p < 0.01, respectively using DMRT.

Compared to the control, exposure to heat stress (30, 35, and 40 °C for 12 and 24 h) led to a significant reduction in chlorophyll *a*, with similar decreasing trends observed for chlorophyll *b* and total chlorophyll content. The most pronounced decline in chlorophyll *a*, *b*, and total chlorophyll content occurred in plants subjected to high temperatures, specifically 40 °C for 24 h, registering decreases of 17.52%, 37.15%, and 23.52%, respectively, compared to the control. Moreover, chlorophyll *b* and total chlorophyll improved significantly (*p* < 0.05) by 34.42% and 23.51%, respectively. Conversely, the total chlorophyll content exhibited a slight increase of 20.41% when seedlings were exposed to salicylic acid alone (0.20 mM). Additionally, the follow-up treatment with SA significantly improved chlorophyll value in all plants exposed to temperature stress. The control plant had a maximum value of chlorophyll content (Fig. 9).

### 3.6 Leaf electrolytic leakage (EL)

**Fig. 10:**
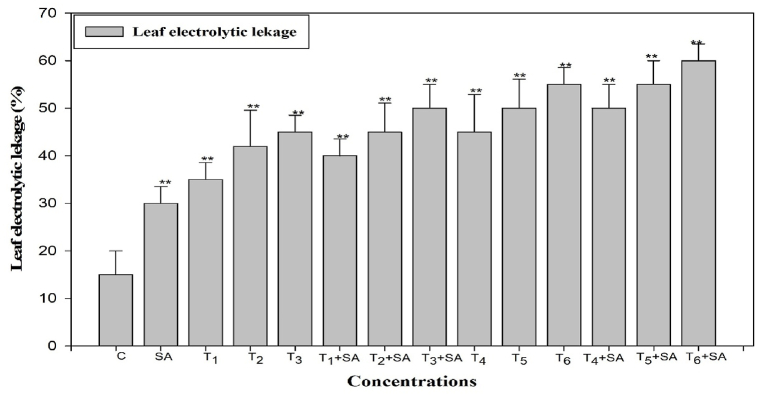
Response of Salicylic acid (0.20 mM) on the high temperature (30, 35 and 40 °C) induced changes on leaf electrolytic leakage in *Trigonella* at 30 days stage if growth [C= Control, SA= Salicylic acid, T_1_ = 30 °C, T_2_ = 35 °C, T_3_ = 40 °C for 12h and T_4_ = 30 °C, T_5_ = 35 °C, T_6_ = 40 °C for 24h of heat stress] Vertical bar shows standard errors and values with (*) and (**) differ significantly at p < 0.05 and p < 0.01, respectively using DMRT.

Electrolyte leakage (EL) serves as an indicator of cell membrane damage and has been utilized to assess heat stress injury. Heat stress notably increased leaf EL by 35.00%, 42.00%, and 54.00% at 30, 35, and 40 °C for 12 h and by 44.00%, 50.00%, and 55.00% at the respective temperatures for 24 h, compared to the control. The interaction of salicylic acid (SA) with temperature treatments also significantly elevated EL levels (*p* < 0.05, 0.01). In contrast, the percentage of heat injury was reduced by 60.00% when SA was applied through spraying on heat-stressed (40 °C) seedlings compared to heat stress alone (Fig. 10).

### 3.7 *Lipid peroxidation*

**Fig. 11:**
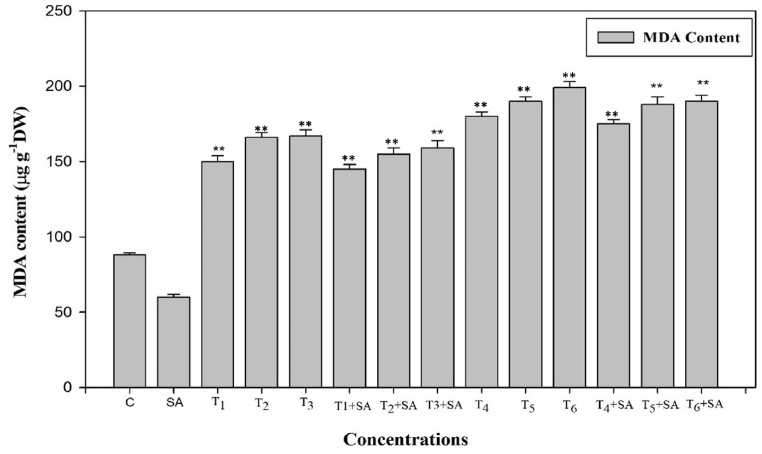
Response of Salicylic acid (0.20 mM) on the high temperature (30, 35 and 40 °C) induced changes on MDA content in *Trigonella* at 30 days stage if growth [C= Control, SA= Salicylic acid, T_1_ = 30 °C, T_2_ = 35 °C, T_3_ = 40 °C for 12h and T_4_ = 30 °C, T_5_ = 35 °C, T_6_ = 40 °C for 24h of heat stress] Vertical bar shows standard errors and values with (*) and (**) differ significantly at p < 0.05 and p < 0.01, respectively using DMRT.

**Fig. 12:**
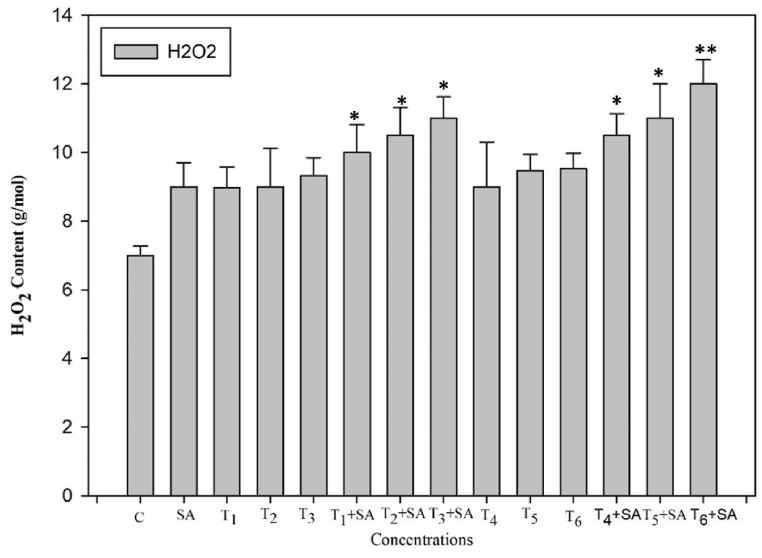
Response of Salicylic acid (0.20 mM) on the high temperature (30, 35 and 40 °C) induced changes on H_2_O_2_ content in *Trigonella* at 30 days stage if growth [C= Control, SA= Salicylic acid, T_1_ = 30 °C, T_2_ = 35 °C, T_3_ = 40 °C for 12h and T_4_ = 30 °C, T_5_ = 35 °C, T_6_ = 40 °C for 24h of heat stress] Vertical bar shows standard errors and values with (*) and (**) differ significantly at p < 0.05 and p < 0.01, respectively using DMRT.

To assess the impact of salicylic acid (SA) on mitigating heat stress-induced oxidative stress, we measured the levels of malondialdehyde (MDA) and hydrogen peroxide (H_2_O_2_) following the application of SA in the presence or absence of high-temperature stress. High temperature stress significantly elevated the MDA and H_2_O_2_ contents as compared with control temperature. When plants exposed to high temperature stress (40 °C for 24h), maximum MDA content was recorded, while the H_2_O_2_ content was increased by 87% at the same amount of stress and duration. Exogenous SA significantly (*p* < 0.05, 0.01) influenced the MDA and H_2_O_2_ under high temperature stress. As a result, the elevated MDA content over the control suggests increased membrane leakiness, reduced heat stability, and higher membrane fluidity (Fig. 11). The relatively lower increase in H_2_O_2_ levels at the minimum temperature indicates reduced cellular toxicity and oxidative damage compared to the control (Fig. 12).

### 3.8 Protein analysis by 2D –GE

**Fig. 13:**
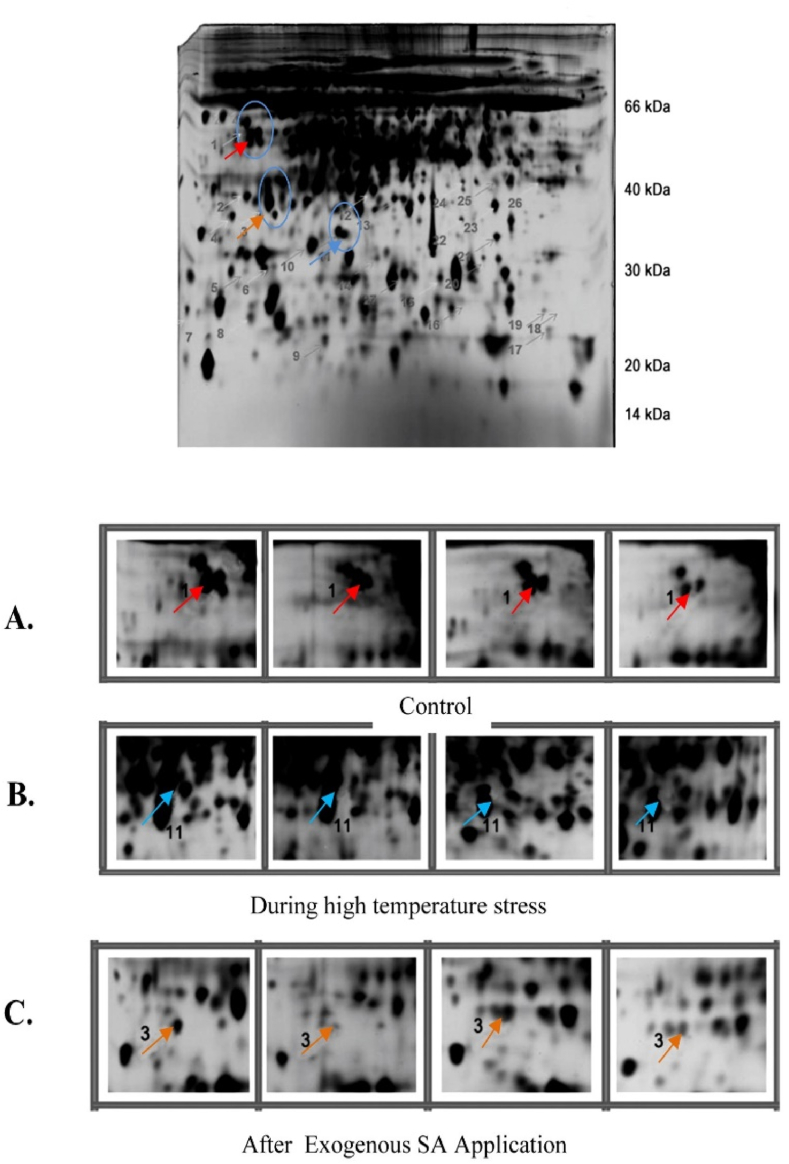
2D gel analysis of proteins isolated from the leaves of *Trigonella foenum-graecum*. In the first dimension (IEF), 150 μg of protein was stacked on a 17-cm IPG strip with a linear gradient of pH 4–7. In the second dimension, 12% SDS-PAGE gels were used. Proteins were visualized by silver staining. The arrows indicate those proteins which have shown differential expression of **(A)** Control, **(B)** During high temperature stress and **(C)** After exogenous SA application.

In heat-stressed fenugreek leaves, we viewed the expression of numerous heat-responsive proteins, identifying over 200 reproducible protein spots on the 2D gel, with 12 exhibiting maximum differential expression. The identification of these differentially expressed protein spots was carried out through MALDI-TOF–TOF/MS, revealing that 4 were upregulated, and 12 were downregulated (Table 1). Notably, after salicylic acid (SA) treatment, protein spots identified as candidates for the photosynthesis-related protein Rubisco carboxylase exhibited a significant increase in fenugreek leaves. The differentially expressed proteins were categorized into six groups based on their mode of expression: (a) proteins downregulated only in control plants but not in treated plants, (b) proteins upregulated in all treated plants, (c) proteins downregulated in all treated plants, (d) proteins downregulated only in control but upregulated in treated plants, (e) proteins absent in the control but present after SA treatment, and (f) proteins assuming different patterns in treated plants compared to other proteins. Analysis of PMF (Probability Mass Function) data of 4 proteins in control derived by MS analysis showed homology to various proteins used in photosynthesis (spots 1, Fig. 13), 4 proteins during high-temperature stress are involved in stress defense (spot 11 Fig. 13) and 4 protein after SA treatment are involved in various physiological functions (spot 3 Fig. 13).

**Table 1:** Expressions of different proteins after SA and high temperature stress.

Spot ID	Gene Bank accession number	Best expect value	Protein PI/Mw (KD)	Protein homologue	Sequence coverage (%)	Amino acid count	Species	Function	Expression
Control									
1a	XP004491629.1	4e-06	6.36/51	Chlorophyll *a*/b binding protein 3 (chloroplast)	42	54	*Trigonella*	Photosynthesis	down regulated
1b	YP007516902.1	8e-12	5.55/48	NADH dehydrogenase (mitochondria	94	45	*Glycine max*	Photosynthesis	down regulated
1c	AFK09931.1	1e-21	6.32/43	Ribulose-1,5 bisphosphate carboxylase oxygenase	32	44	*Trigonella*	Photosynthesis	down regulated
1d	XP004491629.1	4e-06	6.36/51	Chlorophyll *a*/b binding protein 3 (chloroplast)	54	39	*Cicer arietinum*	Photosynthesis	down regulated
**During High Temperature**									
11a	CAA101132	4e-07	6.40/25	Superoxide dismutase	54	47	*Cicer arietinum*	Stress	up regulated
11b	AD245555.1	5e-25	6.15/40	Catalase	100	77	*Vigna radiata*	Stress	up regulated
11c	ACL80571.1	6e-14	5.65/56	Glutathione-S- transferase	45	78	*Glycine max*	Stress	up regulated
11d	NP001237819.1	8e-25	5.95/12	Translationally controlled tumor protein	34	45	*Glycine max*	Stress	up regulated
**After SA application**									
3a	AFHS6428.1	3e-34	5.30/28	Rubisco carboxylase	102	87	*Trigonella*	Photosynthesis	down regulated
3b	AAF15336	1e-08	6.70/36	Cytochrome oxidase subunit 2	34	43	*Faba bean*	oxidation	down regulated
3c	CDF63910.1	1e-15	5.65/28	Maturase (chloroplast)	90	42	*Fabaceae-SPMR-2013*	Transcription	down regulated
3d	XP003548246	1e-21	4.50/25	Enolase like	80	93	*Glycine max*	Glycolytic Pathways	down regulated

## 4 Discussion

High temperature stress significantly impacts the photosynthetic system and the activities of various enzymes associated with metabolic pathways. Previous studies have highlighted that the exogenous application of elicitors can enhance the tolerance mechanisms of various crops under different abiotic stresses [[60], [61], [62]]. Salicylic acid, in particular, plays a crucial role in various physiological and biochemical functions within plants, assuming a pivotal role in enhancing their ability to cope with high-temperature stress [63]. The undertaken research aimed to provide insights into the mechanisms induced by salicylic acid for the protection of growth, photosynthesis, and biochemical parameters against high temperatures (30, 35, 40 °C for 12 h and 24 h) stress in fenugreek seedlings. In this study, it was observed that oxidative damage occurs in fenugreek plants at higher temperatures (40 °C) and that the levels of damage were maximum for 24 h of treatment. This concurs with the work [64] described in which they used the same assay in maize. Certain studies have documented the detrimental effects of salicylic acid (SA) on the germination and seedling growth of crops [65], whereas others, have noted its positive influence on physiological and biochemical parameters [66]. In our experiment, the application of SA + HS resulted in enhancements in growth, physiological and biochemical parameters. The regulatory role of salicylic acid (SA) in stress responses has been found to be dependent on its concentration [67].

High-temperature stress significantly reduced plant growth as measured in terms of germination (%), root and shoot length, fresh mass, and dry mass of roots and shoots (Fig. 1, Fig. 2, Fig. 3). These observations conform with *Vigna radiata* [68], Rice [69], *Morus alba* [70], *Medicago sativa* [71] and *Panicum miliaceum* [72]. The diverse growth responses observed in fenugreek may be attributed to the varied regulation of growth processes at the genetic, biochemical, and physiological levels. High-temperature stress is seen to reduce cell division and elongation [73], primarily due to temperature-stimulated creation of reactive oxygen species [74], suppression of cytoplasmic enzymes, turgor loss, and hormonal imbalance [75], all of which will naturally harm plant growth and ultimately yield. Furthermore, elevated temperatures diminish plant growth by impacting shoot net assimilation rates, consequently affecting the overall dry weight of the plant [76].

Plants are widely recognized for their ability to trigger effective numerous multiplex antioxidant mechanisms to protect themselves from oxidative damage. The antioxidative defence systems includes both non-enzymatic (such as proline) and enzymatic components (such as SOD, CAT, POD, and GR) components to scavenge reactive oxygen species (ROS) and reduce its deleterious effect. The ROS generated through high temperature, can harm the plasma membrane, which then allows vital cytoplasmic components to leak out and ultimately results in cell death [76]. Though, the plasma membrane serves several important functions in plant cells, including generating a selective barrier that allows a cell to be identified and offering an adequate atmosphere for integral proteins to operate. Heat stress induced a notable increase in enzymatic activities compared to the control temperature, indicating that plants undergo oxidative stress under such challenging conditions. These findings parallel the earlier findings in different crops such as *B. oleracea* [77], Tomato [78], cucumber [79], *Capsicum frutescens* [80] and Mung bean [81]. Among all other enzymes, SOD plays the most crucial role in protecting cellular membrane integrity by efficiently scavenging reactive oxygen species (ROS). It initiates the primary defense mechanism of the antioxidant system by converting O2^−^ into H_2_O_2_, which is promptly detoxified by CAT, altering it into H_2_O and O_2_ and thereafter, CAT and POD [78]. CAT and SOD are the enzymes that exhibit the highest level of activity in response to environmental stress conditions. Another crucial antioxidant enzyme is glutathione reductase (GR), responsible for converting glutathione disulfide (GSSG) into reduced glutathione (GSH). This enzymatic activity is essential in maintaining optimal GSH levels, cellular redox state, and protecting cells from the detrimental effects of reactive oxygen species (ROS) [82]. The collective actions of all antioxidant enzymes enhance the efficacy of scavenging, thereby increasing antioxidant functions and contributing to the protection of cellular membranes when subjected to combined pretreatment with salicylic acid and heat stress.

Protein content showed a remarkable increase at higher temperatures, and maximum protein content was found at 40 °C (24h), and SA enhanced the protein content at the same dose (Fig. 6). The protein belongs to this non-speciﬁc defence system against high-temperature stress to act as a modulator against heat shock. Analysis of transcript profiles revealed an increased expression level of numerous genes encoding heat shock proteins (Hsps) following a brief period of oxidative stress treatment [83]. A rise in protein content during stress has been found earlier by Ref. [84].

The findings of the current research suggest a reduction in chlorophyll contents in plants subjected to heat stress (HS). This decrease can be linked to the production of reactive oxygen species (ROS) [85] and lipid peroxidation (LPO) of chloroplast membranes [86]. Heat stress led to a decline in photosynthetic pigments, including chlorophyll *a*, chlorophyll *b*, total chlorophyll (a + b), and carotenoids. However, the introduction of exogenous salicylic acid (SA) to heat-stressed plants enhanced chlorophyll content in fenugreek, which could be indicative of heat stress tolerance [87]. This improvement may be associated with SA protecting chlorophyll from heat-induced degradation or preventing the inhibition of enzymes involved in chlorophyll biosynthesis. These results align with earlier reports in different plant species under various stress conditions [71,88–91].

High-temperature stress is typically exceedingly damaging to enzymatic reactions and biochemical pathways, as well as detrimental diffusion practices and photochemical reactions [2,92,93]. Heat stress decreases thermal stability by damaging the photosystem II and the reactions of the Calvin cycle. Reduction in thermostability with increased temperatures has been reported by Refs. [[88], [89], [90], [91], [92]]. Increased temperatures decrease the stability of membrane lipids and proteins [93]. The reliability of the plasma membrane in such adverse environments is dependent on fast healing and/or restoration of the membrane [94]. The present analysis revealed an increased leaf electrolyte leakage in leaves exposed to heat stress. The highest leaf electrolyte leakage was observed at higher temperatures (40 °C, 24 h). However, the treatment of stressed plants with salicylic acid (SA) enhanced the values for leaf electrolyte leakage. This reaction by SA might be attributed to its contribution to ATPase pump activation [95]. The alteration of membrane fluidity by increasing the amounts of saturated fatty acids, a well-established mechanism for the acclimatization of membranes to heat, could be the reason for the increased membrane fluidity observed with SA application [[96], [97], [98]].

**Fig. 14:**
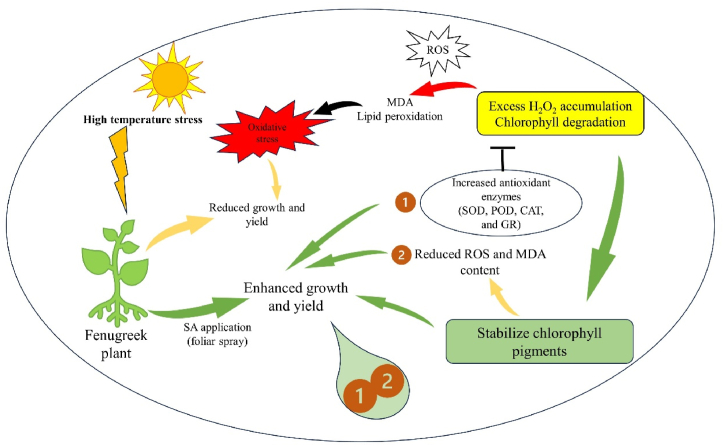
The various mechanisms of foliar salicylic acid (SA) mediated heat-tolerance in fenugreek plants. SOD, superoxide dismutase; POD, peroxidase; CAT, catalase; GR, glutathione reductase; ROS, reactive oxygen species; MDA, malonaldehyde.

Malondialdehyde (MDA) serves as a marker for cellular lipid peroxidation, and the production of reactive oxygen species (ROS) as a result of lipid peroxidation can be harmful to membrane lipids, proteins, and DNA. In this study, it was observed that heat stress led to a significant increase in ion leakage and lipid peroxidation, potentially linked to the excessive generation and accumulation of ROS. Under heat stress in seedlings, there was a sharp increase in MDA concentrations, indicating severe membrane damage due to lipid peroxidation. Plants experiencing heat stress undergo various internal changes, such as increased evapotranspiration leading to dehydration stress [99], along with modifications in associated metabolic functions [100], thereby reducing the integrity of the cell membrane [101]. The increased lipid peroxidation and ROS levels under high-temperature stress suggest a severe impact on cell membrane functionality and integrity [102]. The rise in H_2_O_2_ levels may be attributed to higher superoxide radical dismutation in the presence of a reductant or elevated synthesis and/or decreased activity of peroxidase (POD) and/or catalase (CAT) [103]. The interconnected factors responsible for heat tolerance in fenugreek are visually represented in Fig. 14.

When subjected to high temperatures, numerous plant species undergo developmental, morphological, physiological, and biochemical changes that can impact productivity, quality, and survival [104,105]. In our study, fenugreek leaves and plants experienced severe injuries when exposed to a temperature stress of 40 °C. Our findings revealed that a total of 12 proteins exhibited maximum differential expression in control plants, treated plants, and after the application of salicylic acid (SA). Among these proteins, 4 (spots 11a, 11b, 11c, and 11d) were associated with stress defense, and their abundance was mostly up-regulated under high-temperature stress. Wu et al. [106] employed proteomic techniques to identify 56 differentially expressed protein spots in maize exposed to SA and ABA, and their findings were consistent with ours, highlighting the roles of these proteins in both photosynthesis and defense responses.

Similarly, Wang et al. [107] reported that foliar supplementation of SA in wheat increased the activities of antioxidant enzymes and modulated tolerance levels under heat and high light stress, aligning with our observations. In comparison to high-temperature stress alone, the exogenous application of SA under high-temperature stress reduced the expression of stress defense-related proteins, suggesting that exogenous SA helped mitigate oxidative stress by reducing ROS accumulation. The proteins identified in the leaves of heat-stressed fenugreek plants were involved in photosynthesis, stress defense and detoxification, protein metabolism (translation, processing, and degradation), amino acid metabolism, carbohydrate metabolism, and energy pathways. These results align with the findings of Rahman et al. [108] under drought stress and Yildiz and Terzi [109] under chromium stress, who also observed the differential expression of related proteins. Our results hold functional relevance for various biological processes in response to temperature stress and provide a novel, global insight into the mechanisms of temperature response in higher plants.

## 5 Conclusion

The results of the current study suggest that exposure to heat stress (specifically, 40 °C for 24 h) led to increased production of reactive oxygen species (ROS), lipid peroxidation (LPO), reduced antioxidant enzyme activities, resulting in diminished growth, related parameters, and yield-related characteristics in fenugreek. However, pre-treatment with exogenous salicylic acid (SA) at a concentration of 0.5 mM demonstrated improvements in growth, leaf electrolytic leakage, membrane thermostability, antioxidant enzyme activities, and an upregulation of various stress-related proteins in response to high-temperature stress. The protection or tolerance against heat stress was achieved through enhanced antioxidant enzyme activity and reduced electrolytic leakage at different heat levels, indicating increased scavenging of ROS to protect the photosynthetic apparatus and aid plants in surviving stressful conditions. Additionally, the differential protein profiling revealed the appearance of numerous unique proteins in response to SA + heat stress, indicating their involvement in the plant's defense mechanism under heat stress.

## Funding

The authors express their gratitude for the support provided by Princess Nourah bint Abdulrahman University Researchers Supporting Project number (PNURSP2024R402) at Princess Nourah bint Abdulrahman University, Riyadh, Saudi Arabia.

## Data availability statement

All data supporting the findings of this study are available within the paper.

## CRediT authorship contribution statement

**Sana Choudhary:** Writing – original draft, Conceptualization. **Towseef Mohsin Bhat:** Software, Investigation, Formal analysis, Data curation. **Khairiah Mubarak Alwutayd:** Writing – review & editing. **Diaa Abd El-Moneim:** Writing – review & editing, Funding acquisition. **Neha Naaz:** Writing – review & editing.

## Declaration of competing interest

The authors declare that they have no known competing financial interests or personal relationships that could have appeared to influence the work reported in this paper.

## Abbreviations used

HSPs: Heat Shock Proteins
SA: Salicylic Acid
DDW: Double Distilled Water
DAS: Days After Sowing
POD: Peroxidase
CAT: Catalase
SOD: Superoxide Dismutase
GR: Glutathione Reductase
MDA: Malondialdehyde
2D-PAGE: Two-Dimensional Polyacrylamide Gel Electrophoresis
MALDI-TOF: Matrix Assisted Laser Desorption Ionization Time of Flight
